# Role of vinegar in cardiovascular health: A narrative review

**DOI:** 10.14440/jbm0033

**Published:** 2025-11-20

**Authors:** Ridah Ijaz, Raymond Haward

**Affiliations:** 1 Department of Internal Medicine, Khyber Girls Medical College, Peshawar, Khyber Pakhtunkhwa 25100, Pakistan; 2 Department of Cardiology, Vydehi Institute of Medical Sciences and Research Centre, Bengaluru 560066, Karnataka, India

**Keywords:** Heart, Vinegar, Cardiovascular disease, Naturopathy

## Abstract

**Background:**

Cardiovascular diseases remain the leading cause of mortality globally, driven largely by modifiable risk factors, such as hyperlipidemia, hypertension, diabetes, and oxidative stress. As conventional pharmaceutical interventions often carry adverse effects and economic burdens, there is growing interest in alternative therapies.

**Objective:**

This narrative review explores vinegar, a traditional remedy with historical and cross-cultural usage, as a potential adjunct in cardiovascular health management. Vinegar contains a rich array of bioactive compounds, including acetic acid, polyphenols, amino acids, melanoidins, and tetramethylpyrazine, which may exert antioxidant, anti-inflammatory, lipid-lowering, antihypertensive, and antithrombotic effects. Preclinical studies in animal models and limited human trials indicate promising outcomes in improving lipid profiles, blood pressure regulation, glycemic control, and endothelial function. Despite these benefits, there is a paucity of large-scale, high-quality human studies to validate vinegar’s clinical efficacy and safety.

**Conclusion:**

This review underscores the potential of vinegar as a functional food with cardioprotective properties and advocates for further rigorous research to establish standardized dosing, isolate active constituents, and assess long-term outcomes in human populations.

## 1. Introduction

Cardiovascular diseases (CVDs) are the leading cause of mortality worldwide, accounting for 17.9 million deaths annually.^1^ The contributing risk factors of CVDs include genetics, unhealthy lifestyles (e.g., imbalanced diet, physical inactivity, alcoholism, and smoking), and environmental factors (e.g., air pollution).^2^ CVDs encompass a wide range of diseases, including coronary artery diseases (CADs), cerebrovascular diseases, peripheral arterial disease, congenital heart disease, rheumatic disease, and deep vein thrombosis.^3^ Among these diseases, CAD is the primary cause of death globally.

The most common underlying cause of CAD, as well as other CVDs, is hyperlipidemia,^4^,^5^ which increases the risk of CVDs by 1.8-fold.^4^ Hyperlipidemia increases the levels of total cholesterol (TC) and triglycerides (TGs) and lowers high-density lipoprotein (HDL) levels, increasing the risk of oxidative stress.^5^,^6^ Subsequently, the oxidative stress leads to vascular endothelial injury (VEI), the proposed primary initiator for developing vascular diseases.^7^ Specifically, the formation of reactive oxygen species (ROS) causes VEI, leading to smooth muscle contraction, platelet activation, aggregation, and thrombosis. In addition to hyperlipidemia, other factors that promote oxidative stress include diabetes, smoking, and hypertension.^6^ Similarly, these factors, as well as an imbalance in the coagulation and fibrinolytic processes, also promote thrombus formation.^8^

Since the activation of the coagulation process can lead to heart disease, several drugs, such as statins, lisinopril, and aspirin, are commonly used to alter the function of platelets, promote fibrinolysis, or control hyperlipidemia.^5^ However, these drugs are frequently associated with adverse effects, including insomnia, hepatotoxicity, and constipation, and their cost may limit accessibility for some patients. Therefore, there is a growing need for alternative therapies that are comparatively more affordable and associated with fewer systemic side effects.

Herbal medicine is the most commonly used alternative treatment.^4^ Plants are rich in bioactive compounds with anti-inflammatory and antioxidant properties, giving them therapeutic potential for various conditions. Among traditional remedies, vinegar is one of the most widely used.^4^,^7^ Vinegar is a fermented, water-soluble acetic acid solution used in all cultures around the world.^4^ In addition to its role as a flavoring and food preservative, it has long been recognized for its therapeutic qualities. Vinegar possesses anti-inflammatory, antibacterial, and antioxidant properties and has shown benefits in managing metabolic conditions, such as obesity, diabetes, and CVDs. Its effectiveness is attributed to numerous bioactive substances, including organic acids, phenolics, melanoidins, peptides, and tetramethylpyrazine. However, many other compounds remain unidentified due to the complexity of fermented microbiota and the raw materials used in vinegar production. Owing to its antioxidant and anti-inflammatory properties, vinegar holds particular promise in the prevention and treatment of CVDs.

This review first provides a general background on herbal medicines and their uses in CVDs, followed by a focused discussion on vinegar. Specifically, its historical use, safety dosing, bioactive compounds, cardiovascular effects, and evidence from previous studies are outlined. Finally, the review highlights future insights into clinical studies that evaluate vinegar’s therapeutic potential in cardiovascular conditions.

## 2. History of herbal and traditional medicines

The earliest record of traditional medicine usage dates back to Mesopotamia around 2600 BCE, describing the importance of more than 1000 plant-derived drugs.^9^ Similar historical records highlight the importance of medicinal extracts in ancient civilizations. For example, 52 natural medicinal formulations are documented in the Chinese materia medica record *Wu Shi Er Bing Fang* (ca. 1100 BCE), while over 800 natural medicinal extracts are listed in the Indian Ayurvedic system (ca. 1000 BCE). Hippocrates, often regarded as the father of medicine, also used phytotherapy, or plant-derived treatment, for various diseases. These practices were passed down through generations, preserving the cultural heritage of herbal medicine.

Since the advent of modern medicine, the use of traditional herbs has declined. However, according to the World Health Organization (WHO), approximately 80% of people in developing countries still rely on herbal medicines due to issues of accessibility to and affordability of modern medicine.^10^,^11^ The active ingredients in herbal medicines are beneficial for treating various conditions. Notably, the WHO Traditional Medicine Centers report that 122 substances are widely used across host nations, of which only 94 plant species serve as their primary source, highlighting both the therapeutic richness and cross-cultural consistency of ethnomedicine.^9^

Despite the numerous successes of employing natural products in drug development, drug discovery activities transitioned from natural products to laboratory bench synthesis in the late 1980s.^9^ This shift was largely driven by the complexity of drug discovery and development based on natural products, which necessitates expensive and highly integrated interdisciplinary techniques. However, in recent years, there has been renewed interest in the consumption of herbal products and natural extracts for the treatment of diverse illnesses.^12^ This resurgence is mainly due to their lower cost, reduced side-effect profile, and greater environmental sustainability compared to synthetic pharmaceuticals, whose production often generates environmentally harmful waste.

Beyond health benefits, the use of herbal products enables people to preserve ancestral traditions and strengthen their connection with nature.^12^ These products are also available in the market without significant barriers. Given their undeniable importance, the natural extracts of plants may soon provide the basis for new therapeutic drugs.^13^ Researchers are actively investigating natural extracts for potential incorporation into clinical treatments,^13^ and efforts are also underway to integrate modern and traditional medicine.^12^ This rise of integrative medicine is particularly relevant for managing chronic illnesses, where herbal therapies may improve the mental and emotional health of patients, reduce reliance on pharmaceutical drugs, and play a role in palliative care for patients with terminal illnesses.^12^

There are numerous categories of traditional medicine, including traditional Chinese medicine (TCM), Iranian medicine, Islamic medicine, Ayurveda, Unani medicine, and homeopathy.^11^ Among these, TCM is the most widely practiced traditional healthcare system globally. A key similarity across these traditional medicines is their reliance on herbal medicines derived from plants for treating various diseases. For example, 70% of the Indian population depends on Ayurvedic treatment, also referred to as Indian herbal treatment, for their primary medical requirements. Notably, China has recently invested many resources in the research and development of Chinese herbal medicine, an investment unparalleled by other traditional medical systems globally. Currently, China exports more than 8000 types of plant-derived products to over 130 nations worldwide.

Despite their importance, herbal medicines face several challenges that hinder their broader integration into modern healthcare.^12^ These include limited clinical evidence, insufficient funding for herbal research, lack of standardized guidelines for clinical use, concerns about drug–drug interactions, and issues related to the quality control of herbal drugs. Addressing these challenges requires greater awareness of the cultural and therapeutic significance of herbal medicine, combined with an emphasis on evidence-based validation. Medical professionals should be educated and trained on the pharmacology and safety concerns of herbal medicines, while adequate funding must be allocated to support high-quality human trials. In addition, closer collaboration between healthcare professionals and herbalists should be encouraged to ensure safe and effective integration into practice. If these measures are adopted, healthcare professionals can easily incorporate herbal medicines into clinical practice, and patients can benefit from them.

## 3. Herbal and traditional medicines in cardiovascular treatment

In addition to general herbal treatments, researchers have investigated specific bioactive compounds in plants for their potential to manage cardiovascular conditions. These compounds exert protective effects by preventing platelet activation, endothelial dysfunction, peroxidation, and the excessive generation of ROS, thereby reducing the risk of thrombus formation and arterial constriction.^9^ For example, *Salvia miltiorrhiza* (red sage or *danshen*) contains salvianolic acid B, which protects against ischemia-reperfusion injury. This condition occurs when restored blood flow after prolonged ischemia leads to an increase in the flow of oxygen, triggering the formation of ROS and eventually causing cell death. Similarly, *Astragalus membranaceus*, a type of TCM, contains astragaloside IV, an antioxidant that reduces free radical formation, malondialdehyde (MDA), and superoxide dismutase activity, thereby improving cardiac function and protecting against ischemia. Moreover, since homocysteine imbalance is a major risk factor for vascular disorders, *danshen* has gained attention for its protective effects. It possesses antihypertensive properties, and evidence from a meta-analysis of 10 randomized controlled trials demonstrated that *danshen*, when combined with conventional antihypertensive drugs, may be effective for treating hypertensive left ventricular hypertrophy.^14^ Similarly, another meta-analysis showed that *danshen* pills combined with aspirin effectively improved lipid profiles and hemorheology in CAD patients.^15^ These studies suggest that herbal medications combined with conventional pharmaceutical drugs may be more effective in treating chronic diseases compared to standard treatment alone.

Garlic, also known as *Allium sativum*, is one of the classical plants in traditional medicine, known for its ability to reduce inflammation, oxidative stress, and hyperlipidemia, thereby effectively preventing atherosclerosis and hypertension.^9^ Owing to these properties, garlic has been widely incorporated into herbal health products to boost immunity and enhance cardiovascular health. In addition to their cardioprotective role, some herbal medicines have shown therapeutic potential in treating arrhythmias. One of the most commonly used herbal drugs in clinical practice is digitalis, derived from foxglove leaves. Digitalis enhances cardiac contractility by inhibiting Na^+^/K^+^ pumps, leading to increased calcium levels. Simultaneously, it decreases heart rate by stimulating vagal activity and reducing atrioventricular conduction, thereby improving arrhythmic conditions. It is also effective in the treatment of acute heart failure in critically ill patients.

These examples show that herbal medicines are effective therapeutic agents in the treatment of cardiovascular conditions. Among the diverse bioactive compounds found in plants, flavonoids, polysaccharides, terpenoids, and saponins stand out as the most effective, exerting beneficial physiological effects in humans.^9^

## 4. Vinegar: A traditional remedy with modern scientific interest

Vinegar has been used for more than 3000 years for flavoring, preservation, and treating medical conditions.^16^ Its history can be traced back to around 3000 BCE, when the ancient Babylonians used it as a preserving agent.^17^ In addition, vinegar was also used for the disinfection of wounds. According to the Malaysian Food Act 1983 and Food Regulation 1985, vinegar is defined as a liquid prepared from alcoholic fermentation followed by acetic fermentation of any suitable food source.^18^ The term “*vinegar*” is derived from the French word “*vinaigre*,” meaning sour wine,^17^ referring to the transformation that occurs when wine is left exposed to air. Historical records also show that Hippocrates prescribed vinegar for medical use in 400 BCE.^19^

European alchemists were well aware of the medicinal properties of vinegar during the Middle Ages. Between 1347 and 1771, several European towns experienced recurrent outbreaks of the plague, which was transmitted by flea-infested rats and claimed nearly 50 million lives.^17^ In 1721, the plague struck French towns with such severity that the burial of the dead became unmanageable, forcing prisoners to handle the highly contagious bodies to prevent further spread. Remarkably, while the majority of inmates perished, four reportedly survived by consuming massive amounts of vinegar macerated with garlic daily. Vinegar is also referenced in both the *Old Testament* and the *New Testament*, where it appears as both a drink and a medicine. In Islamic history, Ibn Sina, also known as Avicenna (980–1037), listed the therapeutic characteristics of vinegar in his book *Al-Qanun fi al-Tibb* (*The Canon of Medicine*), describing its properties for coagulation, headache relief, expectoration, and the treatment of burns and skin inflammation.^20^ Its reputation extended into alchemical traditions as well—Bacillus Valentinus (1565–1624) wrote in his book that vinegar was indispensable for alchemical practices.^17^

Based on the raw materials used, vinegars are generally classified into grain vinegar and fruit vinegar.^16^ In European countries, fruit vinegars, such as apple cider vinegar, grape vinegar, and sherry vinegar, are commonly produced through liquid-state fermentation methods. In contrast, Asian countries predominantly manufacture grain vinegars, including rice vinegar, sorghum vinegar, and bran vinegar, using solid-state fermentation methods. Beyond its culinary role as a flavoring agent, vinegar also serves as an effective preservative and medicinal substance.

## 5. Preparation of vinegar

Vinegar production is a two-step bioprocess that produces an acetic acid solution.^21^ In the first stage, yeast converts fermentable carbohydrates into ethanol. The second stage involves the aerobic oxidation of ethanol to acetic acid by acetic acid bacteria. During fermentation, these acids gradually transform into a non-toxic slime, sometimes known as the “mother.” The slime appears as a dark, hazy material in unfiltered vinegar and is developed from naturally occurring pectin and residues. It manifests as a webbed shape or protein molecules joined in strand-like chains. The presence of these slimes indicates high-quality vinegar, as it suggests that the best portion of the substrate remains intact. Traditional vinegar production employs natural vinegar as the starter culture and requires lengthy fermentation periods of up to 1 month, whereas industrial processes can produce vinegar within a single day.

Vinegar production mainly follows two approaches: the traditional method and the industrial method.^21^ The traditional method is a laborious and slow fermentation technique, empirically regulated to promote microbial development and vinegar component accumulation. In contrast, the industrial method is a rapid and mechanized process that relies on submerged fermentation. Although the industrial method is more efficient, it produces vinegar with a weaker aroma compared to the traditional method, as esterases have insufficient time to exert their full effect.

**Figure 1:**
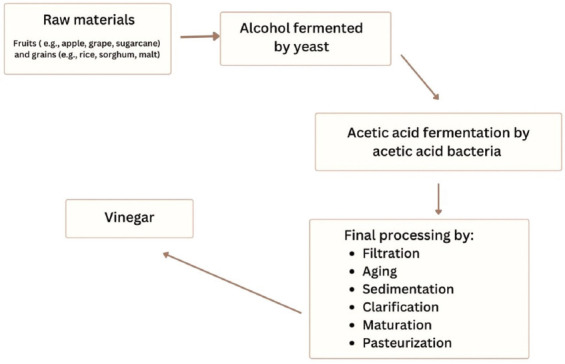
Process of vinegar production.

Although traditional techniques, such as solid-state fermentation and surface culture, produce high-quality final products, they are limited by low efficiency, long processing times, and poor control of operating conditions.^22^ These drawbacks have prompted the utilization of the submerged culture system, a faster and more efficient method widely used in Western and European countries. Regardless of the production method, the production of vinegar is impossible without acetic acid bacteria, which are aerobic bacteria that can produce multiple compounds, particularly acetic acid and other organic acids (Figure 1).

## 6. Bioactive compounds in vinegar and their physiological effects

The bioactive compounds in vinegar exert numerous physiological effects on the human body. Its active ingredients include acetic acid, saccharides, minerals, amino acids, vitamins, organic acids, melanoidins, tetramethylpyrazine, and polyphenols.^16^

### 6.1. Amino acids

In addition to providing essential nutrients that regulate cellular metabolism, amino acids in vinegar act as bioactive substances that enhance immunity and support brain development.^16^ These amino acids contribute to the synthesis of complex peptides such as immunoglobulins, carrier proteins, and neurotransmitters, and when absorbed, they exhibit biological and metabolic properties comparable to free amino acids. Specific amino acids, including histidine, methionine, cysteine, tryptophan, and tyrosine, demonstrate potent antioxidant properties. In addition, during the fermentation of acetic acid in vinegar production, γ-aminobutyric acid (GABA), a non-protein amino acid, is generated. GABA is an essential inhibitory neurotransmitter in the brain. It exerts calming and anti-anxiety properties, enhances cerebral blood flow, improves cellular metabolism, reduces blood pressure, lowers cholesterol level, and promotes diuresis.

During vinegar fermentation, microbial activity breaks down high-molecular-weight proteins into peptides and amino acids.^16^ Sun *et al*.^23^ demonstrated that peptides in Zhenjiang aromatic vinegar, identified through molecular docking and integrating peptidomic analysis, exhibited angiotensin I-converting enzyme (ACE) inhibitory activity, suggesting potential antihypertensive properties of natural constituents in vinegar. In summary, amino acids in vinegar regulate cell metabolism, possess antioxidant properties, and improve immunity and brain function.

### 6.2. Vitamins and minerals

The primary vitamins reported in vinegar are Vitamins B and C.^16^ Vinegar also contains 20 minerals: Na, Mg, K, Ca, Cr, Mn, Fe, Co, Ni, Cu, Zn, As, Se, Sr, Mo, Cd, Sn, Sb, Ba, and Pb. Among the B vitamins, niacin (Vitamin B3) plays an important role in promoting cholesterol excretion and vasodilation. Vitamin C contributes antioxidant activity by preventing oxidative stress and lipid peroxidation. Furthermore, minerals such as K and Zn can generate alkaline compounds that help maintain acid–base balance and prevent blood acidification. Collectively, the vitamins and minerals in vinegar can meet the body’s daily nutritional requirements, which are essential for fostering growth and development and regulating physiological processes.

### 6.3. Polyphenols

Flavonoids and phenolics in vinegar possess anti-inflammatory and antioxidant properties.^16^ By transferring electrons, they neutralize superoxide anion and hydroxyl-free radicals, preventing the chain reactions of oxidative damage. In addition, they chelate metal ions, preventing further redox reaction. These actions help alleviate oxidative stress, regulate lipid metabolism and blood pressure, and protect against aging, CVDs, and hepatic diseases. Researchers have found that the antioxidant activity of vinegar increases with longer aging time, and that traditionally brewed vinegar demonstrates stronger antioxidant activity than industrially produced vinegar.

### 6.4. Organic acids

Organic acids in vinegar are classified as either volatile (e.g., acetic acid, butyric acid, and propionic acid) or non-volatile (e.g., malic acid, citric acid, and succinic acid).^16^ These compounds originate from the fermentation process and the raw ingredients used. Among them, acetic acid and lactic acid are the two most reported organic acids in vinegar.

Organic acids play a vital role in regulating cellular metabolism. For example, acetic acid exhibits cardioprotective effects by lowering TC and serum TG levels through enhanced fecal bile acid excretion and hepatic lipogenesis inhibition, thereby effectively treating hyperlipidemia and aiding in weight loss.^16^ It has been shown to reduce body fat by increasing fatty oxidation enzymes and to decrease renin and angiotensin levels in the treatment of hypertension. In addition, organic acids display antibacterial properties and improve insulin sensitivity in type 2 diabetic patients.

A study has shown that polysaccharides and organic acids in *Monascus* vinegar improved hyperlipidemia in high-fat diet-fed mice by reducing inflammatory markers, preventing hepatocyte lipid accumulation, and modulating lipid metabolic pathways.^24^ Similarly, another study compared the effects of *Monascus* vinegar and acetic acid vinegar in treating hyperlipidemia and hyperglycemia.^25^ The study showed that although both types possessed hypoglycemic and hypolipidemic properties, in *Monascus* vinegar, acetic acid played a key role in regulating diabetes and improving lipid profiles.

In summary, organic acids in vinegar are effective agents in controlling hyperlipidemia, hyperglycemia, bacterial growth, and obesity prevention.

### 6.5. Sugars

Vinegar contains variable amounts of sugars, including monosaccharides, disaccharides, and polysaccharides, which contribute to anticoagulation, antioxidation, and immunomodulation.^16^ A study by Sun *et al*.^26^ showed that polysaccharides extracted from buckwheat vinegar demonstrated strong antioxidant properties due to the total reducing power, hydroxyl-free radical scavenging, and 1-diphenyl-2-picrylhydrazyl radical scavenging. Similarly, another study demonstrated that alditols and monosaccharides in sorghum vinegar inhibited cyclooxygenase-1 and thromboxane-A2 synthase, leading to reduced platelet aggregation, a mechanism with potential benefits for CVDs prevention and treatment.^27^ In summary, sugars in vinegar contribute significantly to its physiological effects.

### 6.6. Melanoidins

The Maillard reaction leads to the formation of melanoidins, which are brown macromolecules derived from sugars and nitrogenous substances (e.g., proteins, peptides, and amino acids).^16^ The primary processes that produce melanoidins in vinegar are heating and aging. In solid-state fermentation, thermal processing breaks down residual proteins, hemicelluloses, starches, and microbial metabolites into reducing sugars and amino acids, which then undergo polymerization through the Maillard reaction to form melanoidins.

Melanoidins contribute not only to the flavor of vinegar but also to its antioxidant and antibacterial properties.^16^ Their antioxidant capacity is enhanced through interactions with phenolic compounds and the formation of intermediate compounds such as heterocyclic compounds and reductones during the Maillard reaction. Melanoidins chelate metal ions, preventing redox reactions and free radical formation. They have also been shown to reduce ROS formation in normal human and mouse livers through a mitophagy-dependent pathway, alleviating oxidative stress *in vivo* and *in vitro*.^28^ Moreover, melanoidins exhibit antibacterial properties through their ability to chelate metal ions.^16^ In addition, melanoidins have an antihypertensive effect.^29^ Since ACE leads to the activation of the renin–angiotensin–aldosterone system, which is the main contributor to the rise in blood pressure, its inhibition is necessary to control blood pressure. A few studies suggest that melanoidins may inhibit ACEs, contributing to the lowering of blood pressure, although current evidence remains limited. In summary, melanoidins in vinegar help protect against oxidative stress, inhibit bacterial replication, and may play a role in regulating blood pressure.

### 6.7. Tetramethylpyrazine

Tetramethylpyrazine, sometimes referred to as ligustrazine, is a typical byproduct of the Maillard process and the fermentation of microorganisms.^16^ Similar to melanoidins, it is also formed during the thermal processing and aging of vinegar.

In addition to contributing to the taste of vinegar, tetramethylpyrazine exerts several physiological benefits, including vasodilation, antioxidation, lipid-lowering effects, and the inhibition of platelet aggregation.^16^ Chen *et al*.^30^ discovered that tetramethylpyrazine extracted from Chinese black vinegar demonstrated hypolipidemic and antioxidant properties in HepG2 cells. These effects were mediated through the activation of peroxisome proliferator-activated receptor gamma–liver X receptor–ATP-binding cassette transporter A1 (PPARγ–LXR–ABCA1) pathway.

Tetramethylpyrazine in vinegar also has potential in alleviating myocardial injury.^31^ It has been demonstrated to lower lactate dehydrogenase and creatine phosphokinase levels, as well as to reduce apoptosis in cardiomyocytes subjected to anoxia-reoxygenation injury, thereby reducing infarct size and preventing further cardiac damage. In addition, tetramethylpyrazine preserves mitochondrial function, thus improving oxygen consumption rate, decreasing ROS generation, reducing mitochondrial permeability, and decreasing cytochrome c release, which collectively improve the function of injured cardiomyocytes and reduce the rate of apoptosis. These cardioprotective effects are attributed to the downregulation of voltage-dependent anion channel 1 expression in the myocardium and its role in mitochondria. In summary, tetramethylpyrazine may be an effective agent in reducing ischemic heart injury.

### 6.8. Summary

The bioactive ingredients in vinegar not only contribute to its flavor but also function as potent antioxidants by preventing DNA peroxidation and inhibiting the formation of ROS.^16^ Consequently, vinegar may reduce aging, abnormal cell proliferation, and tumor formation while enhancing the body’s antioxidant defense mechanism. In addition, these substances have demonstrated potential in improving overall cardiac function and lipid profile, thereby positioning vinegar as an effective agent in treating cardiovascular conditions.

## 7. Safety and dosing of vinegar

### 7.1. Recommended dose

Regarding dosing, it is necessary to consume vinegar at a level that does not lead to harmful side effects. Commercial vinegar has an acetic concentration of 4–7%.^32^ Notably, an acetic acid concentration of >20% can cause caustic injury to the esophagus. Therefore, the safe amount of commercial vinegar is two tablespoons per meal.

### 7.2. Adverse effect on humans

When consumed in unsafe amounts, vinegar may cause hypokalemia, inflammation of the oropharynx, caustic injury to the esophagus, and gastrointestinal side effects such as flatulence, reflux, burping, and altered bowel activity.^32^ There have been limited studies regarding the safe dosage of vinegar. For example, a study involving 155 obese Japanese patients showed that 15 mL (750 mg acetic acid) or 30 mL (1500 mg acetic acid) of vinegar did not cause any adverse effect.^33^ In contrast, a study indicated that long-term consumption of 250 mL of apple cider vinegar for 6 years led to hypokalemia in a female patient.^34^ Moreover, a single tablespoon of rice vinegar was reported to cause second-degree caustic injury to the esophagus and inflammation of the oropharynx in a female who consumed it to dislodge a piece of crab shell; she recovered spontaneously within a few days.^35^ Patients with type 1 diabetes who consumed apple cider vinegar were reported to experience increased hypoglycemic episodes due to delayed gastric emptying.^36^ Other side effects linked to apple cider vinegar include flatulence, burping, and constipation.^36^

In summary, although vinegar provides numerous physiological benefits, excessive consumption may cause a few systemic side effects. Therefore, caution is warranted when consuming vinegar.

### 7.3. Adverse effects on animals

Scientific research has used various animal models, including diabetic, obese, high-fat diet, or cholesterol-rich diet animals, to study the effects of vinegar dosages.^36^ Although many of them were experimented with high doses of vinegar, most animal studies have not reported any adverse effects or lethality.

## 8. Cardiovascular effects of vinegar

Vinegar has various cardiovascular effects on the body. Although studies remain limited, existing evidence supports its cardioprotective role and potential implications in treating CVDs. While the exact mechanisms are still unknown, several theories have been proposed to explain the various physiological effects of vinegar in CVDs.

For example, vinegar may increase nitrite and nitrate levels.^34^,^37^ Polyphenols in vinegar activate nitric oxide (NO) synthase, thereby increasing NO levels and leading to smooth muscle relaxation. NO plays a central role in cardiovascular protection through its vasodilatory, antioxidant, and antithrombotic properties.^35^,^36^,^38^,^39^ It inhibits platelet aggregation and adhesion, preventing thrombus formation, while also downregulating leukocyte adhesion molecules, thereby preventing leukocyte recruitment and its interaction with platelets.^35^,^38^ In addition, NO suppresses the growth of vascular smooth muscle cells, a critical stage in thrombus formation through a cyclic guanosine monophosphate-dependent mechanism.^35^,^36^,^38^,^39^

NO also exhibits antioxidant effects. It reduces low-density lipoprotein (LDL) radicals and lowers tissue factor expression, thus attenuating coagulation.^37^,^40^ In addition, NO also reduces the levels of ROS.^36^,^39^ Although the exact processes of antioxidant mechanisms of NO remain unclear, a proposed mechanism is that NO inhibits nicotinamide adenine dinucleotide phosphate oxidase enzymes, thereby reducing oxidation. Another proposed explanation is that NO stimulates the nuclear factor erythroid 2-related factor 2 (NRF2) pathway, initiating the transcription of antioxidant genes, including those of superoxide dismutase, catalase, glutathione peroxidase, and thioredoxins. Furthermore, hypertension also elevates vascular xanthine oxidoreductase activity, which acts as a nitrite reductase enzyme and facilitates the production of NO from nitrite rather than promoting the formation of ROS. These antioxidant effects of NO decrease matrix metallopeptidase 2 activity, thereby mitigating hypertension-induced vascular remodeling. They also attenuate cardiac remodeling by inhibiting the angiotensin II-induced tumor growth factor-beta signaling pathway, thereby improving cardiac function. Overall, NO confers extensive vasoprotective properties, and vinegar may enhance these benefits. However, further studies are needed to clarify the effects of vinegar on NO levels to confirm its clinical efficacy.

Vinegar also has a role in improving lipid profiles, which are key mediators in the development of atherosclerosis. Its components effectively reduce C-reactive protein (CRP), fibrinogen, apolipoprotein B/apolipoprotein A ratio, factor VII, oxidized LDL, MDA, and atherosclerotic lesions in the aorta.^34^,^37^ Dietary acetic acid in vinegar increases fecal bile acid excretion, reducing lipid levels.^38^,^41^ A hypothesized mechanism is that vinegar suppresses ATP lyase, fatty acid synthase, and 3-hydroxy-3-methylglutaryl-coenzyme A reductase, causing decreased fatty acid synthesis.

Given that low-grade inflammation is a risk factor for the development of CVDs, therapeutic strategies to lower it are essential.^39^,^42^ The immune system’s components, particularly the innate immune system and lymphocytes, interact with platelets, causing thrombus formation.^40^,^43^ One of the most significant risk factors of inflammation is an imbalanced diet.^39^,^42^ A study showed that participants consuming two tablespoons of vinegar with meals daily for 4 weeks exhibited a significant reduction in two pro-inflammatory biomarkers, interleukin-1 and interleukin-12.^39^,^42^ In contrast, there were no effects on the levels of other pro-inflammatory or anti-inflammatory biomarkers. Notably, although the CRP levels were not statistically significant, they tended to increase across subjects. These findings suggest that vinegar may be an effective anti-inflammatory treatment; however, further research is needed to confirm its anti-inflammatory properties.

VEI is linked to CAD, as damaged endothelial cells release ROS, which promote apoptosis, vasomotor contraction, platelet adhesion, and platelet aggregation, leading to thrombus formation.^7^ Damaged endothelial cells also release protein kinase zeta (PKCς), a key factor that plays a crucial role in cellular metabolism, growth, and differentiation. A decreased level of PKCς reduces cellular injury and oxidative stress in vascular smooth muscle cells. A study has shown that Hengshun aromatic vinegar, a type of grain vinegar, downregulated PKCς, thereby preventing VEI and reducing apoptosis through the inhibition of caspases, B-cell lymphoma 2-associated X protein, p53, and protein inhibitor of activated STAT Y, thus enhancing antioxidant capacity.^7^ In addition, Henghshun aromatic vinegar reduced the levels of homocysteine, endothelin, LDL, and MDA while increasing the levels of NO, NO synthase, glutathione, and glutathione peroxidases, thereby alleviating cellular injury and oxidative stress.

Over the past few decades, due to an unhealthy diet and lack of physical activity, there has been a trend of increased prevalence of obesity at an alarming rate. Obesity is a significant contributor to diabetes, hyperlipidemia, hypertension, and CVD.^41^,^44^ In addition, it leads to the development of obesity-induced cardiomyopathy through mechanisms such as altered cardiac metabolism, inflammation, oxidative stress, excessive visceral adiposity, cardiac fibrosis, and hypertrophy, ultimately leading to structural remodeling of the heart. A study on obese rats showed that fruit vinegar reduced plasma adiponectin and visceral adipose tissue levels, suggesting its potential as an effective intervention for obesity-related cardiovascular complications, as well as highlighting its anti-inflammatory and anti-adiposity properties.

Fibrinolysis effectively prevents thrombus formation by activating plasmin, an enzyme that degrades fibrin and clotting factors.^42^,^45^ Plasmin generation is mainly mediated by tissue plasminogen activator and urokinase-type plasminogen activator. Tissue plasminogen activator inhibitor 1 (PAI-1) regulates both processes involved in plasmin production. It is predominantly expressed in vascular endothelial cells across various organs as well as in adipocytes. Elevated levels of PAI-1 are linked to CVDs and contribute to infection and inflammation. Vinegar has been shown to effectively lower PAI-1 levels, leading to controlled coagulation. In particular, a study showed that the ethanol extract of Kurozu Moromi, a type of Japanese black vinegar, lowered PAI-1 levels, effectively increasing plasmin levels and promoting fibrinolysis.^42^,^45^

Vinegar may also be an effective antihypertensive agent.^43^,^46^ Acetic acid, the principal component of vinegar, has been shown to effectively reduce blood pressure by inhibiting ACE activity and regulating renin levels, significantly reducing both systolic and diastolic blood pressure. In addition, it affects calcium absorption, which further contributes to blood pressure regulation. Hence, vinegar shows potential as an effective agent in regulating blood pressure.

**Figure 2:**
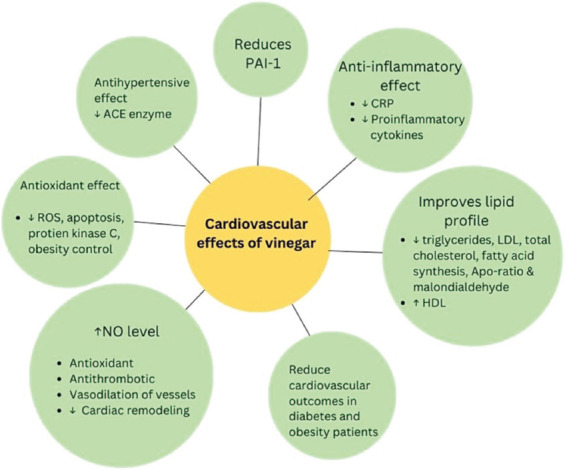
Cardiovascular effects of vinegar Abbreviations: ACE: Angiotensin I-converting enzyme; Apo: Apolipoprotein; CRP: C-reactive protein; HDL: High-density lipoprotein; LDL: Low-density lipoprotein; NO: Nitric oxide; PAI-1: Plasminogen activator inhibitor 1; ROS: Reactive oxygen species.

Vinegar may also be an effective agent for preventing cardiometabolic risk factors in diabetic patients. As uncontrolled blood glucose levels can lead to CVDs, which remain a leading cause of death in diabetic patients, effective glycemic management is essential.^44^,^47^ Significant interventions for preventing CVDs include lifestyle modification and controlling blood glucose levels. A meta-analysis demonstrated that apple cider vinegar consumption significantly reduced cholesterol and TG levels in diabetic patients.^45^,^48^ It also improved glycemic control by reducing hemoglobin A1c (HbA1c) and postprandial glucose levels. Although the precise mechanism is unknown, proposed pathways include delayed gastric emptying, inhibition of lipogenesis, stimulation of lipolysis, reduced hepatic glucose production, enhanced glucose utilization, and improved insulin secretion. By improving both glucose levels and lipid profiles, vinegar may reduce the likelihood of CVDs^44^,^45^,^47^,^48^ (Figure 2).

These studies suggest that vinegar may have therapeutic potential in treating CVDs. However, more clinical studies should be conducted to confirm its cardiovascular efficacy.

## 9. Clinical and experimental studies of vinegar and cardiovascular health in humans and animals

There are limited studies on the cardiovascular effects of vinegar in humans. However, animal studies suggest that vinegar may support cardiovascular health. When high-cholesterol diet-fed rats were administered coconut vinegar for eight weeks, there was a significant reduction in TG, fasting blood glucose, and LDL levels, accompanied by a significant increase in HDL levels.^46^,^49^ Furthermore, coconut vinegar significantly improved hepatic cholesterol and TG levels, reduced lipid peroxidation, and hence enhanced liver function. In the aorta, it also increased NO bioavailability and inhibited lipid peroxidation.

Similarly, another study showed that when high-fat diet-fed Wistar rats were treated with Schidandra fruit vinegar, there was a significant reduction in body weight, LDL, TG, TC, MDA, free fatty acid, aspartate aminotransferase, and alanine aminotransferase, accompanied by a significant increase in the content of HDL and the activity of superoxide dismutase.^47^,^50^ These effects were attributed to an increase in the expression of β-oxidation-related proteins, such as PPARα, carnitine palmitoyltransferase 1, and peroxisomal acyl-coenzyme A oxidase 1, as well as the modulation of antioxidant pathway regulators, such as Kelch-like ECH-associated protein 1, NRF2, and heme oxygenase 1.

In addition to unhealthy diets and obesity, disruption of gut microbiota is another mechanism contributing to dyslipidemia.^48^,^51^ Obesity alters gut microbiota, significantly contributing to steatosis, dyslipidemia, and inflammation. Fruit vinegar may promote beneficial bacteria, thereby improving lipid profiles. It was shown that rats fed with *Rosa roxburghii* fruit vinegar exhibited increased levels of beneficial bacteria (e.g., Proteobacteria, Bacteroidetes, Lactobacillaceae, *Bacteroides*, and *Akkermansia*) and decreased levels of harmful bacteria (e.g., Ruminococcaceae, Erysipelotrichaceae, Ruminococcaceae _UCG-013, Lachnospiraceae, *Allobaculum*, and Actinobacteria). Actinobacteria and Firmicutes were responsible for dyslipidemia, whereas *Bacteroides* improved the lipid profile. These findings indicate that modulation of gut microbiota by fruit vinegar may represent a potential strategy for managing dyslipidemia and obesity.

Food processing may enhance the overall quality of vinegar.^49^,^52^ For example, ultrasound treatment of hawthorn vinegar, along with increased concentration, significantly boosted its antioxidant capacity, making it more effective than pasteurized or untreated vinegar.^49^,^52^ This increased antioxidant capacity improved lipid profiles in Wistar-Albino rats. These findings highlight the importance of vinegar processing methods and concentration in maximizing the beneficial outcomes of vinegar.

A combination of vinegar with different herbal medicines may enhance its potential in treating cardiovascular conditions. For example, liquid garlic extract with various vinegars improved antioxidant capacity, lipid profile, and liver and kidney functions in hypercholesterolemic obese rats.^4^ These improvements may be attributed to the increase in bioactive substances provided by the garlic liquid extract–vinegar combination. Similarly, another study showed that a mixture of *Zinger officinale*, *A. sativum*, *Citrus limon*, honey, and *Malus domestica* vinegar extracts significantly improved lipid profiles and reduced body weight in high-cholesterol diet-fed rats compared to the control group.^5^

Many studies have experimentally modified animals, such as cholesterol-fed, diabetic, obese, and high-fat diet-fed animals.^33^,^36^ However, most of these studies did not report any adverse events, whether related to the experimental manipulation or the administration of vinegar. Among humans, only a few studies have demonstrated the cardiovascular efficacy of vinegar, and these studies have several limitations. Research on 120 obese Lebanese students found that apple cider vinegar consumption over 12 weeks significantly reduced anthropometric variables, including body weight, body mass index (BMI), and waist-to-hip ratio, and improved blood glucose, cholesterol, and TG levels.^50^,^53^ In contrast, a meta-analysis of 33 clinical trials found no significant decreases in BMI, body fat, LDL, and cholesterol.^51^,^54^ However, there were significant decreases in fasting blood glucose, cholesterol, TGs, and HbA1c in diabetic patients. Another meta-analysis by Hadi *et al*.^45^,^48^ showed that for patients with type 2 diabetes, daily apple cider vinegar significantly reduced plasma glucose, HbA1c, TC, and TGs levels. However, no significant effects were observed in HDL, LDL, and fasting insulin levels. Similarly, a meta-analysis of 16 trials found that acetic acid in vinegar significantly lowered fasting blood glucose levels in diabetic patients and TG levels across healthy, obese, and diabetic patients.^52^,^55^ The study showed no effect on LDL, HDL, HbA1c, and anthropometric variables, such as BMI and body weight.

These findings suggest that acetic acid in vinegar has a potential advantage solely for obese and type 2 diabetic patients. However, the meta-analyses had notable limitations, including a small number of trials, high risk of bias, poor study designs, small sample sizes, and inadequate research into vinegar’s cardiovascular effects. Given the potential of vinegar in treating CVDs, further studies are needed to establish its clinical efficacy.

## 10. Potential risks and limitations of the vinegar theory

Although vinegar exhibits numerous cardiovascular effects, there are limited studies demonstrating its use in clinical practice or confirming its cardiovascular benefits on humans. While some studies are available, their small sample sizes and methodological limitations hinder a detailed assessment of vinegar’s cardiovascular efficacy. Moreover, although active ingredients such as acetic acid and polyphenols are primarily responsible for these effects, relatively limited studies have investigated the impact of these individual active ingredients alone.

## 11. Conclusion

Vinegar, a traditional remedy with a long history of culinary and medicinal use, contains diverse bioactive compounds—such as acetic acid, polyphenols, amino acids, melanoidins, and tetramethylpyrazine—that collectively exhibit antioxidant, anti-inflammatory, antihypertensive, lipid-lowering, and antithrombotic effects. Preclinical animal studies consistently demonstrate improvements in lipid metabolism, glycemic control, endothelial function, and blood pressure regulation, while limited human trials suggest modest benefits, particularly in populations with obesity and type 2 diabetes. Despite these promising findings, current evidence is constrained by small sample sizes, short intervention periods, heterogeneity in study design, and a lack of standardized dosing.

Therefore, while vinegar may be considered a functional food with potential cardioprotective properties, its clinical application remains preliminary. Future research should prioritize well-designed, large-scale randomized controlled trials to establish efficacy, safety, optimal dosage, and the role of individual bioactive components. Rigorous investigation into long-term outcomes will be essential before vinegar can be confidently integrated into evidence-based cardiovascular prevention and management strategies.

## Data Availability

Not applicable.
